# Case Report: Co-occurrence of Myocarditis and Thrombotic Microangiopathy Limited to the Heart in a COVID-19 Patient

**DOI:** 10.3389/fcvm.2021.695010

**Published:** 2021-07-29

**Authors:** Thomas Menter, Nadine Cueni, Eva Caroline Gebhard, Alexandar Tzankov

**Affiliations:** ^1^Department of Pathology, Institute of Medical Genetics and Pathology, University Hospital Basel, University of Basel, Basel, Switzerland; ^2^Intensive Care Unit, University Hospital Basel, Basel, Switzerland

**Keywords:** COVID-19, heart, myocarditis acute and fulminant, thrombus, thromboinflammation

## Abstract

We report on an impressive case of a previously healthy 47-year-old female Caucasian SARS-CoV-2 positive patient who died within 48 h after initial cardiac symptoms. Autopsy revealed necrotizing myocarditis and extensive microthrombosis as the cause of death. The interesting feature of this case is the combination of both myocarditis and extensive localized microthrombosis of cardiac capillaries. Microthrombosis was not present in other organs, and the patient did not show typical features of diffuse alveolar damage in the lungs. Taken together, our morphologic findings illustrate the angiocentric, microangiopathic, thromboinflammatory disease with significant thrombotic diathesis prevalent in COVID-19, which has been previously described in the literature, likely warranting thromboprophylaxis even in oligosymptomatic circumstances. This case also delineates several potential etiologies for microthrombosis, i.e., inflammatory reactions and primary hypercoagulative states. Further systematic analyses on risk stratification for receipt of prophylactic anticoagulation in COVID-19 are urgently required.

## Introduction

Based on early empiric evidence and autopsy observations (1–3), anticoagulation has emerged as an important topic in treatment of COVID-19 (4). It is now well-established that thromboses significantly contribute to disease burden in COVID-19 and thus warrant immediate attention by treating physicians regardless of disease severity (5, 6). Myocarditis is also rare but nevertheless an acknowledged comorbidity in COVID-19 (7). Few cases have been examined by histopathology so far, and they showed divergent features ranging from subtle inflammatory infiltrates not fulfilling diagnostic criteria for borderline myocarditis to overt necrotizing inflammation (8–10).

Here, we present a case of a previously healthy patient positive for SARS-CoV-2 who died of cardiac complications consisting of necrotizing myocarditis and extensive microthrombosis within 48 h after initial cardiac symptoms.

## Case

A 47-year-old female suffered from oligosymptomatic flu-like disease for a week before she was found unconscious and apneic at home. Advanced cardiac life support was promptly administered by paramedics. Upon hospital admission, electrocardiography (ECG) showed ST-segment depression in all the leads and elevations in augmented vector right (aVR). Subsequently performed coronary angiography revealed no relevant coronary stenosis. Echocardiography detected moderately reduced left ventricular function (left ventricular ejection fraction (LVEF) 30%) and normal right ventricular size and function. There was no evidence of left ventricular hypertrophy or dilatation. Computerized tomography excluded pulmonary thromboembolism but showed bilateral lower-lobe consolidations. High-sensitivity troponin T (hsTropT) was elevated (272 ng/l at admission; peak of 507 ng/l 10 h after admission), as were brain natriuretic peptide (>70,000 ng/l) and C-reactive protein (282 mg/l). The leucocyte count was within normal range. A nasopharyngeal swab was positive for SARS-CoV-2. Apart from mild thrombocytopenia (140 G/L) and mildly prolonged activated partial thromboplastin time (aPTT) (39 s) at the time of admission, all other coagulation parameters were within normal ranges. Despite exhaustive invasive intensive care interventions including continuous adrenalin/noradrenaline infusion, prophylactic antibiotic therapy (piperacillin/tazobactam), and two further attempts of cardiac resuscitation, the patient died of cardio-respiratory failure within 48 h of admission. Her detailed clinical course is shown in the timeline section. Previous clinical history included episodes of depression, which had been treated with venlafaxine, and a cholecystectomy.

**Figure 1:**
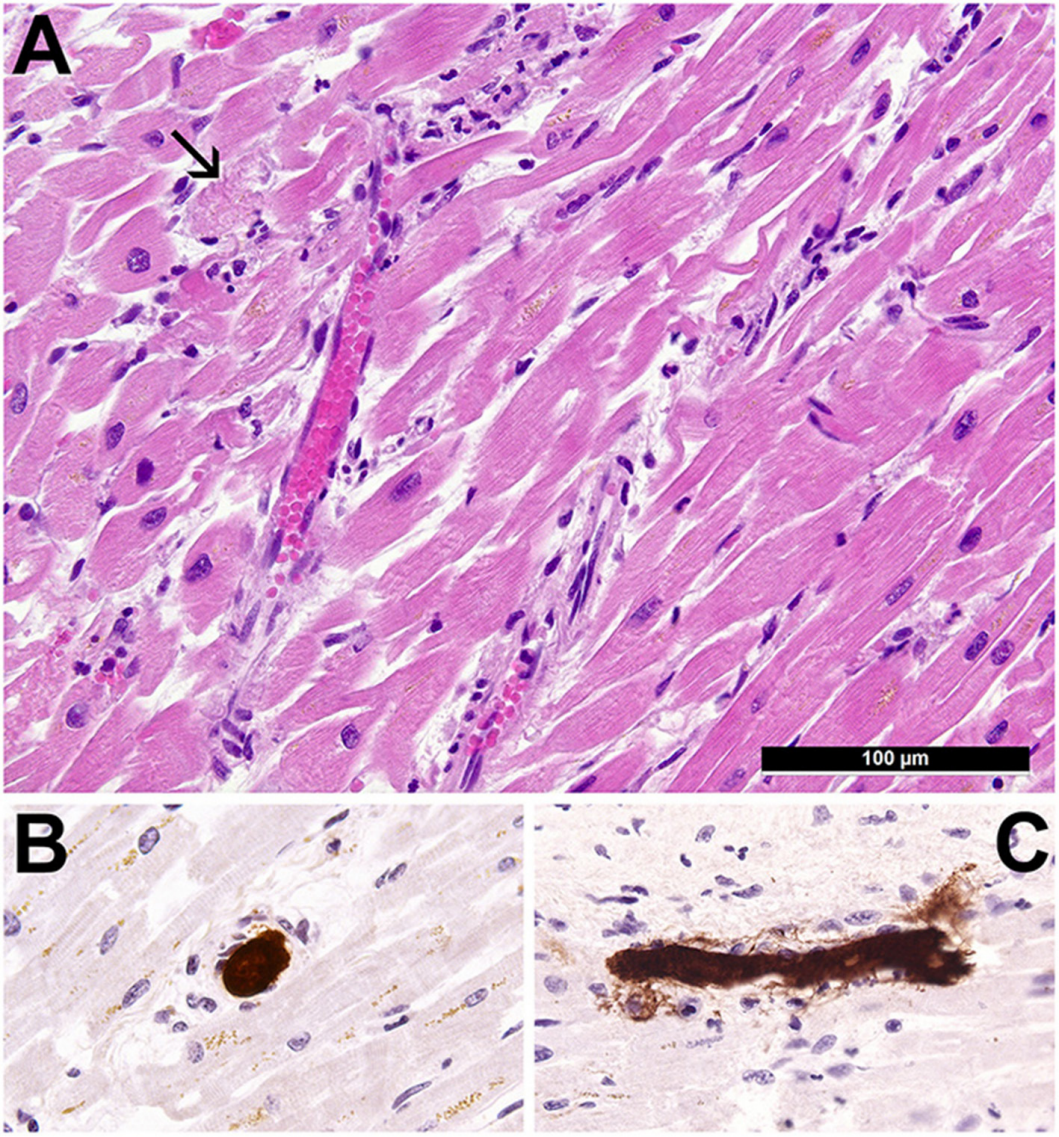
Cardiopathological findings. **(A)** Morphology of the heart showing multifocal inflammatory infiltrates consisting of neutrophilic granulocytes, lymphocytes and histiocytes, capillarostasis, and perifocal single-cell necroses of cardiomyocytes (arrow) (H&E, ×400). **(B,C)** Immunohistochemical staining for fibrin demonstrating cross section and longitudinal section of capillaries with prominent microthrombi occluding the lumens (immunohistochemistry for fibrin, ×400).

An autopsy was performed. No relevant comorbidities apart from obesity [body mass index (BMI) 31.6] were noticed. Major findings included moderate bilateral suppurative pneumonia with COVID-19-characteristic capillary stasis, yet without diffuse alveolar damage. Most notably, the heart presented as normotrophic and irregularly perfused with mild diffuse necrotizing myocarditis (Figure 1A) accompanied by extensive thrombotic microangiopathy of cardiac capillaries (Figures 1B,C; microthrombi in cardiac capillaries immunohistochemically stained for fibrin), which was determined as the cause of death. RT-qPCR of heart tissue was positive for SARS-CoV-2-*N*-gene (Ct 35.7). Immunohistochemistry for adenovirus was negative.

## Timeline

**Table:** 

**Date**	**Event**
1 week before admission to hospital	Flu-like symptoms with symptomatic treatment (SARS-CoV-2 test negative)
Day 1	Advanced cardiac life support due to asystole (after at least 10 min without basic life support measurements) with return of spontaneous circulation after 10 min
	Referral to the emergency department of a tertiary care center (SARS-CoV-2 test positive)
	Diagnostics: moderate left ventricular ejection dysfunction (LVEF 30%), lower pulmonary lobe opacities, no evidence of coronary artery disease or thrombosis, nor pulmonary embolisms or pneumothorax, normal electrolyte values
Day 2	Admission to intensive care unit requiring mechanical ventilation and with multiorgan failure
	One episode of ventricular fibrillation treated with defibrillation
	Extracorporeal membrane oxygenation not performed, as the time period between first reanimation attempts was prolonged, inducing severe hypoxic encephalopathy with extensively elevated neuron-specific enolase in the absence of hemolysis
Day 3	Death after renewed unsuccessful reanimation for 20 min

## Discussion

The interesting feature of this COVID-19 case study is the combination of both myocarditis and extensive microthrombosis of cardiac capillaries.

Our group, amongst others, has previously presented comprehensive autopsy cohorts of patients succumbing to COVID-19 describing microthrombosis predominantly in the lungs and further organs (2, 11, 12). Microthrombi in the pulmonary capillary bed and subsequently increased intravascular pressure in the pulmonary circulation have been attributed to heart failure in several studies. A recent report focusing on heart pathology described microthrombi in 12/15 COVID-19 cases (13); thrombi were also found in some control cases with influenza infection, metastatic carcinoma, or advanced severe bacterial pneumonia. In line with this case, we have previously investigated cardiopathological characteristics of patients succumbing to COVID-19 associated respiratory failure, similarly demonstrating a high incidence of capillary dilatation, stasis, and microthrombosis, especially in cases with detectable SARS-CoV-2 cardiac viral load (14).

It is well-acknowledged that COVID-19 predisposes to a procoagulatory state. The underlying pathophysiology for thromboinflammation is likely multifaceted, involving direct endothelial damage by SARS-CoV-2, secondary inflammatory endothelial damage, an overexpression of procoagulatory genes (e.g., *SERPINE* genes) in target organs, and generation of neutrophilic extracellular traps, and immunological, particularly antiphospholipid-mediated processes [rev. in (15)].

Myocarditis, in particular borderline myocarditis, in COVID-19 patients has been described (7). In a systematic review of 41 studies compiling 316 cases of COVID-19 autopsies, Roshdy et al. identified five cases (i.e., 1.5%) with inflammatory infiltrates fulfilling the Dallas criteria of myocarditis (8). In ≈10% of cases, mild focal inflammatory infiltrates in the myocardial interstitium had been noticed. In one study on endomyocardial biopsies taken for elucidating the cause of acute heart failure or in suspicion of myocarditis (16), isolated cases showed both presence of SARS-CoV-2 genomes and inflammatory infiltrates also affecting small vessels, while thrombi had not been described in this series.

In our case, the patient exclusively presented with localized cardiac microthrombi; other organs were not affected (also confirmed by immunohistochemistry). Microthrombi were partially associated with inflammatory infiltrates and single-cell cardiomyocyte necrosis, which we interpret as a sequela of the thrombotic microangiopathy. Acute heart failure, which is the non-disputable cause of death, can be attributed to both features—myocarditis and microthrombi—and our findings strongly support that both morphological features can be collectively interpreted as a rare but severe COVID-19-related thromboinflammatory cardiac complication. There was no evidence of a preexisting heart condition based on imaging and autopsy findings as well as the clinical history of the patient. Admittedly, it has to be considered that her intake of antidepressant (venlafaxine) might have contributed to cardiac pathology, yet the features described in single case reports and a review article of individuals treated with venlafaxine and cardiac problems were not evident in this case (17–19). Furthermore, there was no evidence of serotonin syndrome, mydriasis, or seizures.

Taken together, our morphologic findings and the preexisting literature illustrate that COVID-19 is an angiocentric, particularly microangiopathic, thromboinflammatory disease with significant thrombotic diathesis, in all likelihood warranting thromboprophylaxis even in oligosymptomatic circumstances. This case also delineates several potential etiologies for microthrombi: inflammatory reaction and primary hypercoagulative state. Further systematic analyses on risk stratification for receipt of prophylactic anticoagulation in COVID-19 are urgently required.

## Patient Perspective

Heart failure is a severe complication of COVID-19. As illustrated in this case, it can also arise in previously healthy patients and might develop independently of pulmonary findings. Myocarditis and microthrombosis of the cardiac capillaries are potentially treatable etiologies of heart failure in such instances, yet their diagnosis may be difficult without histological examination. Thorough investigation of both coagulation parameters and the myocardium might be therefore required in patients with unexplained or rapidly deteriorating heart failure in the setting of COVID-19. It remains to be determined if prophylactic anticoagulation even in oligosymptomatic COVID-19 patients is feasible.

## Data Availability Statement

The raw data supporting the conclusions of this article will be made available by the authors, without undue reservation.

## Ethics Statement

Ethical review and approval was not required for the study on human participants in accordance with the local legislation and institutional requirements. The patients/participants provided their written informed consent to participate in this study.

## Author Contributions

AT performed the autopsy. AT and TM designed the study and wrote the manuscript. NC and EG took care of the patient and provided clinical data. All authors contributed to the article and approved the submitted version.

## Conflict of Interest

The authors declare that the research was conducted in the absence of any commercial or financial relationships that could be construed as a potential conflict of interest.

## Publisher's Note

All claims expressed in this article are solely those of the authors and do not necessarily represent those of their affiliated organizations, or those of the publisher, the editors and the reviewers. Any product that may be evaluated in this article, or claim that may be made by its manufacturer, is not guaranteed or endorsed by the publisher.
